# GloPL, a global data base on pollen limitation of plant reproduction

**DOI:** 10.1038/sdata.2018.249

**Published:** 2018-11-20

**Authors:** J. M. Bennett, J. A. Steets, W. Durka, J. C. Vamosi, G. Arceo-Gómez, M. Burd, L. A. Burkle, A. G. Ellis, L. Freitas, J. Li, J. G. Rodger, M. Wolowski, J. Xia, T-L. Ashman, T. M. Knight

**Affiliations:** 1 Institute of Biology, Martin Luther University Halle-Wittenberg, Am Kirchtor 1, 06108, Halle (Saale), Germany; 2 German Centre for Integrative Biodiversity Research (iDiv) Halle-Jena-Leipzig, Deutscher Platz 5e, 04103 Leipzig, Germany; 3 Department of Plant Biology, Ecology and Evolution, Oklahoma State University, Stillwater, OK, USA; 4 Illumination Works, 2689 Commons Blvd. Suite 120, Beavercreek, OH 45431, USA; 5 Department of Biology, Case Western Reserve University Cleveland, Ohio 44106-7080, USA; 6 Department of Community Ecology, Helmholtz Centre for Environmental Research – UFZ, Theodor-Lieser-Straße 4, 06120 Halle (Saale), Germany; 7 Department of Biological Sciences, University of Calgary, Calgary, AB, Canada; 8 Department of Biological Sciences, Eastern Tennessee State University, Johnson City, TN, USA; 9 School of Biological Sciences, Monash University, Melbourne VIC 3800, Australia; 10 Department of Ecology, Montana State University, Bozeman, MT 59715 USA; 11 Department of Botany and Zoology, University of Stellenbosch, Private Bag X1, Matieland 7602, South Africa; 12 Rio de Janeiro Botanical Garden, Rua Pacheco Leão 915, Rio de Janeiro - RJ, 22460-030, Brazil; 13 Taizhou University, Jiaojiang District, Taizhou City, Zhejiang, P. R. China; 14 Department of Plant Ecology and Evolution, Evolutionary Biology Centre, Uppsala University, Norbyvägen 18 D, SE-75236 Uppsala, Sweden; 15 Institute of Natural Sciences, Federal University of Alfenas, Gabriel Monteiro da Silva street 700, Alfenas, Minas Gerais, 37130-001, Brazil; 16 College of Life Sciences, South-Central University for Nationalities, Wuhan, Hubei, P. R. China; 17 Department of Biological Sciences, University of Pittsburgh, Pittsburgh, PA 15260, USA

**Keywords:** Pollination, Plant ecology

## Abstract

Plant reproduction relies on transfer of pollen from anthers to stigmas, and the majority of flowering plants depend on biotic or abiotic agents for this transfer. A key metric for characterizing if pollen receipt is insufficient for reproduction is pollen limitation, which is assessed by pollen supplementation experiments. In a pollen supplementation experiment, fruit or seed production by flowers exposed to natural pollination is compared to that following hand pollination either by pollen supplementation (i.e. manual outcross pollen addition without bagging) or manual outcrossing of bagged flowers, which excludes natural pollination. The GloPL database brings together data from 2969 unique pollen supplementation experiments reported in 927 publications published from 1981 to 2015, allowing assessment of the strength and variability of pollen limitation in 1265 wild plant species across all biomes and geographic regions globally. The GloPL database will be updated and curated with the aim of enabling the continued study of pollen limitation in natural ecosystems and highlighting significant gaps in our understanding of pollen limitation.

## Background & Summary

Plants rely on abiotic and/or biotic agents to transport pollen grains to ovules for sexual reproduction. An inadequate quantity or quality of pollen can reduce plant reproductive success. Thus, it is valuable to understand whether, and how strongly, seed production is limited by pollination. The magnitude of pollen limitation is estimated from hand pollination experiments^1^; if plants hand-pollinated with outcross pollen produce more seeds than plants in a naturally pollinated treatment, then reproduction is limited by aspects of pollen receipt rather than by abiotic resources. Thus, the difference in reproductive output between supplemented and naturally pollinated groups can be used to calculate an effect size metric that reflects pollination sufficiency.

**Figure 1:**
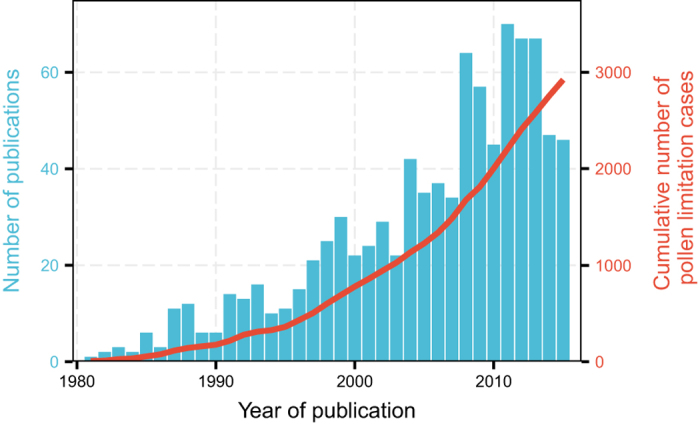
The distribution of year of publication of studies that measured pollen-limitation of plant reproduction shown in blue and the accumulative number of cases per year, shown in red, in the GloPL database.

Understanding the causes and consequences of pollen limitation is an active and accelerating area of research^2^ (Fig. 1) and many central questions remain. For example, it is still not known to what extent pollen limitation represents temporal variability in either pollen receipt or resource availability^3–5^ or is a consequence of adaptations (e.g. ovule number, reward size) to stochastic pollination environments^6–8^. Furthermore, reports of global pollinator declines adds urgency to the key question of whether anthropogenic perturbations in the pollination or resource environment are increasing levels of pollen limitation^9^. On global scales, land use change, climate change, and species invasions are altering the interactions between native pollinators and plants which may affect plant reproduction^10–15^. Pollen limitation has ecological and evolutionary consequences that cascade through populations, communities and ecosystems. In particular, pollen limitation affects reproduction, a key demographic vital rate, that in turn influences population growth^4,16^, community structure^17,18^ and ecosystem functioning^4,15^. It also contributes to natural selection on floral traits through female fecundity^19–21^. Thus, pollen limitation of plant reproduction is important for the abundance and distribution of wild plant species, necessitating the analysis of underlying causes of its variation and potential consequences.

**Figure 2:**
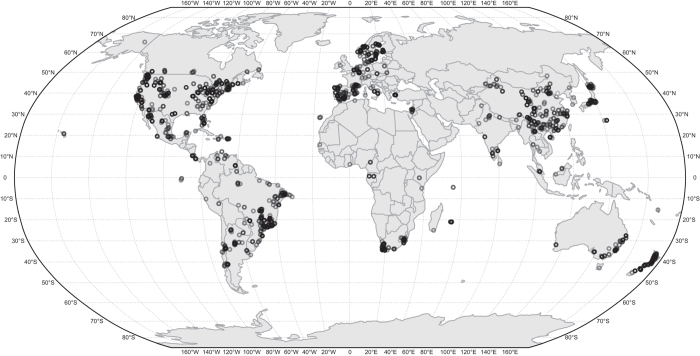
Global distribution of data from the GloPL database. Dots represent the location of each case in the dataset.

Here we present the GloPL database that brings together data from 927 unique publications that conducted hand pollination experiments, allowing calculation of 2969 values of pollen-limitation effect size for 1265 wild plant species across the globe (Fig. 2). Each row of data represents a unique experiment with hand pollination and natural pollination treatments, sometimes including multiple response variables for female fecundity (e.g., proportion of flowers setting fruit, proportion of ovules setting seeds, seeds per flower, seeds per fruit, and seeds per plant). Across these studies, all response variables are positively correlated to each other (*r* range from 0.38 to 0.98; all *P* < 0.0001), as seen in previous syntheses^2^. Overall, effect sizes estimated from the proportion of ovules setting seeds were lower than other response variables (*P* < 0.0001). Salient features of the experimental design or data analysis, for example, level at which the hand-pollination supplementation was applied (i.e., to a single flower, branch, inflorescence, or entire plant), sample sizes and standard deviations, are also included.

**Figure 3:**
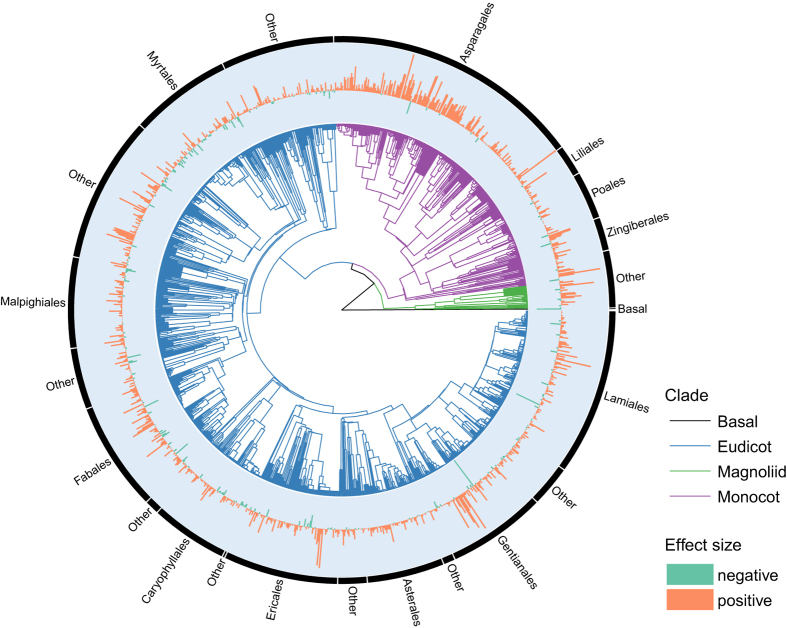
Phylogenetic distribution of data from the GloPL database. Tree structure is derived from taxonomy. Pollen-limitation effect size is given for each species in a bar plot, where orange bars indicate a positive effect size and blue bars indicate an effect size of zero or below (i.e. no pollen limitation). Major angiosperm groups are denoted.

**Figure 4:**
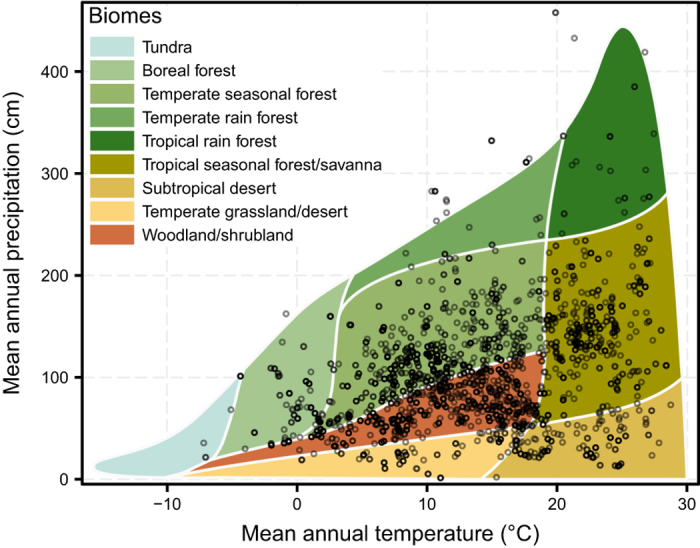
The distribution of species’ locations in GloPL database across the nine major global terrestrial biomes. Terrestrial biomes as described by Whittaker^31^ are defined by mean annual temperature (x axis) and mean annual precipitation (y axis). Temperature and precipitation data was extracted for the GloPL localities from the Chelsa database^32^.

The GloPL database can be used to assess how biogeographic and evolutionary history, as well as local contemporary environmental factors, affect the magnitude of pollen limitation. This database provides unprecedented wide geographic (Fig. 2) and phylogenetic (Fig. 3) coverage, as well as representing all major terrestrial biomes (Fig. 4). The database contains 0.5% of all angiosperm species, with representatives from both core (e.g. Magnoliids, Monocotyledons, eudicots) and basal (i.e. Nymphaeaceae) groups and a third of all flowering plant families distributed across three quarters of all currently recognized orders (APG IV; Fig. 3). However, there are gaps in the representation of some lineages, and these pinpoint areas in need of more research, e.g. Magnoliids (0.28% sampled species) and the basal angiosperms (i.e., ANA grade; only one species *Nymphaea ampla* out of ~200 known species) are poorly sampled. The geographic areas that are understudied include several recognized biodiversity hotspots (e.g., tropical Africa and south east Asia; Fig. 2), as well as some biomes (e.g., tundra, temperate and tropical forests; Fig. 4).

The GloPL database will be maintained and curated with the aim of enabling the continued study of pollen limitation in natural ecosystems and to highlight gaps in our understanding of pollen limitation. Updates to GloPL will be available at https://idata.idiv.de/.

## Methods

A global and temporally deep database on pollen limitation was assembled as part of a working group funded by sDiv, the Synthesis Centre of the German Centre for Integrative Biodiversity Research (iDiv) Halle-Jena-Leipzig. This database expands that used in Knight *et al.* (2005), which covered published studies from 1981 to 2003, to include studies from 2004 to 2015 (see Data_citations.xlsx, Data Citation 1). In addition, reliable studies not listed in ISI but published or unpublished (e.g., theses or data sets) in China, South Africa, and Brazil from 1981 to 2015 were added, including those in the respective national languages.

### Literature search

We searched the literature for published hand pollination experiments using ISI’s Web of Science and Biological Abstracts and the keywords ‘‘pollen limit^∗^’’, ‘‘supplement^∗^ poll^∗^’’, and ‘‘hand poll^∗^’’. Additional search engines and terms were used for searching Chinese literature (CNKI database [1999 to 2016, http://oversea.cnki.net] and Wanfang database [1981–2016, http://www.wanfangdata.com] with the terms “pollinator limitation”, “hand-pollination”, “mating system”, “breeding system”, “sexual system”, “seed-set”, “reproductive ecology”, “pollination ecology”) and South American (mainly Brazilian) literature in the Scielo database (http://www.scielo.org) (with the terms ‘‘hand poll ecology’) and South American (mainly Brazilian) literature in the Scielo database’’, “reproductive biology”, “reproduct* system”, “breeding system”, “mating system”, “compatib ecology’) and South American (mainly Brazilian) literature in the Scielo database”, “pollinatecology”) and South American (mainly Brazilian) literature in the Scielo database”). Additionally, the journal Darwiniana was checked manually for the years 1996 to 2003, which were not in Scielo. To extract South African studies, the location key words (Cape, Fynbos, Renosterveld, Karoo, CFR, GCFR, South Africa) were used in combination with the additional keywords “pollinat^∗^”, “breeding system” and “mating system”. Theses collections were searched at the Bolus Library at the University of Cape Town. Unpublished data was provided by J. A. Steets, T.-L. Ashman, A. Iler, T. M. Knight, J. G. Rodger, and J. Wright.

We included cases that measured at least one of the following five response variables of reproductive output: fruit set (proportion of flowers setting fruit), proportion of ovules setting seed (seed-set or seed:ovule ratio), number of seeds per fruit, number of seeds per flower, and number of seeds per plant. We report, the sample size, mean, and a measure of variance (with the exception of binomially distributed variable fruit set, for which variance could be estimated from the mean and sample size)^22^. Data published in graphical form were digitized using Plot digitizer (available at: http://www.southalabama.edu/colleges/artsandsci/physics/software.html).

For each case, we recorded data from a single pollen supplementation experiment with a control and supplemented pollination treatment. The control treatment is exposed to natural pollination, whereas the hand pollination treatment is either pollen supplementation (i.e. manual outcross pollen addition without bagging, also allowing for natural pollination; 69% of records) or manual outcrossing of bagged flowers (31%). Although, bagging can effect seed production (e.g., by changing the microclimate around bagged flowers etc.) not considering data from bagged experiments excluded a large amount of data and disproportionately excluded data from some regions, e.g., China and South America. We also recorded whether the treatments were applied at the level of the flower, inflorescence, partial plant (ramet, branch), or whole plant, and which response variables were measured. Plants that were manipulated in other ways (i.e. emasculation and outcrossed hand pollinated) are denoted as such. We treated each year and population as a separate case (i.e., row in the database). Data was also inputted as a separate case when multiple time-periods (e.g., season) or multiple morphs (e.g., flower color, gender) within a population were sampled and when additional treatments were conducted (e.g., nutrient addition).

### Pollen-limitation effect size metric

**Figure 5:**
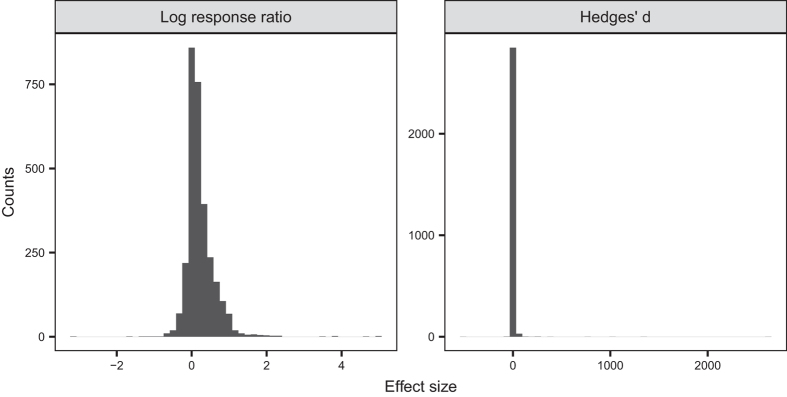
Histograms showing the distribution of the pollen limitation effect size when calculated as a log response ratio and Hedge’s D (range −535 to 2599).

A unified single pollen-limitation effect size was calculated for each record. Preference was given to the least biased response variable (i.e., seeds per plant), which is also the most appropriate for questions concerning ecological or evolutionary dynamics^23^. When this variable was not given but multiple other response variables were provided, priority was given in the following order: seeds per flower, seeds per fruit, fruit set, and proportion of ovules setting seeds. The type of response variable that was used in the pollen-limitation effect size metric is denoted for each record. We calculated the magnitude of the pollen-limitation effect (PL_Effect_Size) as the log response ratio:

$$\mathrm{PL}\_\mathrm{Effect}\_\mathrm{Size}=\ln\left[\left(X_{\mathrm{hand}}\right)/\left(X_{\mathrm{natural}}\right)\right]$$

where X is the mean of the response variable. However, the log response ratio does not produce an estimate of effect size for cases with a zero event in either the numerator or denominator. It such cases it is generally not recommended to add a constant to the numerator and denominator to calculate the log response ratio, because this can result in a greatly over-estimated effect size^24^. However, in our case, adding a constant likely underestimates the true log response ratio in most cases and did not produce unusually high values in the context of this dataset. One alternative to adding a constant in calculation of log odds ratio is using Hedges D ratio^25,26^ as an effect size, which for this dataset was not worthwhile because it showed poor statistical properties (Fig. 5). Another alternative would be to omit all cases with zeros but as zero responses occurred most frequently for natural pollination in this dataset, signifying high pollen limitation, excluding them would cause us to underestimate total pollen limitation more than adding a constant. We thus chose to add a constant (0.5) to both treatments when one or both had a zero response, as this was better than the alternatives. To facilitate, sensitivity analysis, where a user compares the results obtained when the constant is added to the results obtained when cases with zero responses are omitted, we have provided a column denoting where a constant was added in order to calculate the effect size.

### Study location, time and biogeographical data sets

Author supplied localities were recorded as geographic coordinates (latitude, longitude) in decimal degrees. When a location description was provided but without geographic coordinates, the coordinates were determined using Google earth images.

### Phylogeny

We used the dated molecular phylogeny created by Zanne and colleagues^27^ on 32,223 land plant species to create a phylogeny for our focal plant species (with first constraining the tree to have Monicots and Eudicots as sister taxa to align more with the modern understanding of plant phylogeny (APG IV 2016). Congeneric species present in our database but missing from this tree were bound into the phylogeny at the genus level using the function congeneric.merge in the pez package of R^28^. For species in our database that were in genera not represented in the Zanne phylogeny (~5% of the species, a full list of the genera that were hand placed on the phylogeny are provided with Data Citation 1), we searched the literature for information about the placement of these species and grafted them into the phylogeny manually. If a phylogeny with branch lengths was found, we carried over the approximate branch lengths. For example, if the node between two genera indicated a length of 20% from the root of the family, branch length from the same two genera were set to be the same in this tree. However, in most cases, only topology could be reliably estimated, in which case the species was grafted in at the half-way point on the branch leading up to its sister-group.

### Code availability

All code used to generate the master effect size and all code used to produce each figure in this manuscript is provided in R programing language^29^ and is open access via github (https://github.com/idiv-biodiversity/pollen-limitation-data-descriptor).

## Data Records

### Data Record 1

The GloPL database is available by downloading ‘GloPL.csv’ (Data Citation 1). Data in GloPL (Data Citation 1) are organized by author last name and publication year. The database includes 2969 experiments from 927 publications, which were conducted on 1265 plant species from 163 families and 45 orders (Figs. 1 and 2). The data are available in both Excel and text formats in Data Dryad (Data Citation 1). Updates to the data and metadata will be curated through the iDiv data portal (https://idata.idiv.de/).

A case is defined as a comparison of the reproductive output (proportion of flowers setting fruit, proportion of ovules setting seeds, seeds per fruit, seeds per flower, seeds per plant) of plants receiving hand pollination with those receiving natural pollination. When studies were replicated across sites and/or years at the same site, each unique site and year combination became one case in the dataset. We treated each year and population as a separate data case (i.e., row in the database), as were time periods within a season or multiple morphs (e.g., flower color, gender) within a population and different experimental treatments (e.g., nutrient addition, herbivore exclusion). For each case within the GloPL database, we provide plant taxonomic information and geographic coordinates (detailed meta-data for each column is located in PL_Meta_data.xlsx, Data Citation 1).

### Data Record 2

The phylogenetic tree of plant species in Data Record 1 is provided in two formats (nexus and .tre) in the files entitled ‘SiteTree_VS’.

## Technical Validation

All entries were checked by a second person, and any record that appeared to be inconsistent was rechecked against the original source. Geographic coordinates were quality checked by projection. Taxonomy was confirmed to be in accordance with the current accepted species names, authority and plant family. We used Taxonomic Name Resolution Service v4.0^30^. In the instances where the published study used a synonym of an accepted species name, we gave the original and revised the name. We consulted The Plant List v1.1 2010 (available at: www.theplantlist.org) for any names for which the TNRS did not offer an opinion.

## Additional information

**How to cite this article**: Bennett, J. M. *et al*. GloPL, a global data base on pollen limitation of plant reproduction. *Sci. Data*. 5:180249 doi: 10.1038/sdata.2018.249 (2018).

**Publisher’s note**: Springer Nature remains neutral with regard to jurisdictional claims in published maps and institutional affiliations.

## Supplementary Material
