# Publisher Correction: GloPL, a global data base on pollen limitation of plant reproduction

**DOI:** 10.1038/s41597-018-0006-1

**Published:** 2019-01-22

**Authors:** J. M. Bennett, J. A. Steets, J. H. Burns, W. Durka, J. C. Vamosi, G. Arceo-Gómez, M. Burd, L. A. Burkle, A. G. Ellis, L. Freitas, J. Li, J. G. Rodger, M. Wolowski, J. Xia, T-L. Ashman, T. M. Knight

**Affiliations:** 1 Institute of Biology, Martin Luther University Halle-Wittenberg, Am Kirchtor 1, 06108 Halle (Saale), Germany; 2 German Centre for Integrative Biodiversity Research (iDiv) Halle-Jena-Leipzig, Deutscher Platz 5e, 04103 Leipzig, Germany; 3 Department of Plant Biology, Ecology and Evolution, Oklahoma State University, Stillwater, OK USA; 4 Illumination Works, 2689 Commons Blvd. Suite 120, Beavercreek, OH 45431 USA; 5 Department of Community Ecology, Helmholtz Centre for Environmental Research – UFZ, Theodor-Lieser-Straße 4, 06120 Halle (Saale), Germany; 6 Department of Biological Sciences, University of Calgary, Calgary, AB Canada; 7 Department of Biological Sciences, Eastern Tennessee State University, Johnson City, TN USA; 8 School of Biological Sciences, Monash University, Melbourne, VIC 3800 Australia; 9 Department of Ecology, Montana State University, Bozeman, MT 59715 USA; 10 Department of Botany and Zoology, University of Stellenbosch, Private Bag X1, Matieland, 7602 South Africa; 11 Rio de Janeiro Botanical Garden, Rua Pacheco Leão 915, Rio de Janeiro - RJ, 22460-030 Brazil; 12 Taizhou University, Jiaojiang District, Taizhou City, Zhejiang P. R. China; 13 Department of Plant Ecology and Evolution, Evolutionary Biology Centre, Uppsala University, Norbyvägen 18 D, SE-75236 Uppsala, Sweden; 14 Institute of Natural Sciences, Federal University of Alfenas, Gabriel Monteiro da Silva street 700, Alfenas, Minas Gerais 37130-001 Brazil; 15 College of Life Sciences, South-Central University for Nationalities, Wuhan, Hubei P. R. China; 16 Department of Biological Sciences, University of Pittsburgh, Pittsburgh, PA 15260 USA; 17 Department of Biology, Case Western Reserve University, Cleveland, Ohio 44106-7080 USA

Correction to: *Scientific Data* 10.1038/sdata.2018.249, published online 20 November 2018

J. H. Burns was omitted in error from the author list of the original version of this Data Descriptor. This omission has now been corrected in both the HTML and PDF versions.

